# The Relationship Between Co-morbidity, Screen-Detection and Outcome in Patients Undergoing Resection for Colorectal Cancer

**DOI:** 10.1007/s00268-021-06079-3

**Published:** 2021-03-27

**Authors:** Mark S. Johnstone, Donald C. McMillan, Paul G. Horgan, David Mansouri

**Affiliations:** Academic Unit of Surgery, University of Glasgow, New Lister Building, Glasgow Royal Infirmary, 8-16 Alexandra Parade, Glasgow, G31 2ER UK

## Abstract

**Background:**

Bowel cancer screening increases early stage disease detection and reduces cancer-specific mortality. We assessed the relationship between co-morbidity, screen-detection and survival in colorectal cancer.

**Methods:**

A retrospective, observational cohort study compared screen-detected (SD) and non-screen-detected (NSD) patients undergoing potentially curative resection (April 2009–March 2011). Co-morbidity was quantified using ASA, Lee and Charlson Indices. Systemic inflammatory response was measured using the neutrophil lymphocyte ratio (NLR). Covariables were compared using crosstabulation and the χ2 test for linear trend. Survival was analysed using Cox Regression.

**Results:**

Of 770 patients, 331 had SD- and 439 NSD-disease. A lower proportion of SD patients had a high ASA (≥3) compared to NSD (27.2% vs 37.3%; *p* = 0.007). There was no significant difference in the proportion of patients with a high (≥2) Lee Index (16.3% SD vs 21.9% NSD; *p* = 0.054) or high (≥3) Charlson Index (22.7% SD vs 26.9% NSD; *p* = 0.181). On univariate analysis, NSD (HR 2.182 (1.594–2.989;*p* < 0.001)), emergency presentation (HR 3.390 (2.401–4.788; *p* < 0.001)), advanced UICC-TNM (III or IV) (*p* < 0.001), high ASA (≥3) (HR 1.857 (1.362–2.532; *p* < 0.001)), high Charlson Index (≥3) (HR 1.800 (1.333–2.432; *p* < 0.001)) and high (≥3) NLR (HR 1.825 (1.363–2.442; *p* < 0.001)) were associated with poorer overall survival (OS). NSD predicted poorer cancer-specific survival (CSS) (HR 2.763 (1.776–4.298; *p* < 0.001)). On multivariate analysis, NSD retained significance as an independent predictor of poorer OS (HR 1.796 (1.224–2.635; *p* = 0.003)) and CSS (HR 1.924 (1.193–3.102; *p* = 0.007)).

**Conclusions:**

Patients with SD cancers have significantly lower ASA scores. After adjusting for ASA, co-morbidity and a broad range of covariables, SD patients retain significantly better OS and CSS.

## Introduction

Colorectal cancer is the fourth most common cancer in the UK, with approximately 41,000 new cases and 16,000 deaths each year [1]. The Scottish Bowel Screening Programme was introduced in 2007 and involved a combined guaiac-based faecal occult blood (gFOBt) and faecal immunochemical test (FIT) followed by colonoscopy for those patients testing positive [2]. More recently that has changed to a quantitative FIT-based programme. Patients aged between 50 and 74 years are invited for screening in Scotland. There is good evidence to suggest that this approach to screening increases the number of early stage cancers diagnosed and reduces cancer-specific mortality [3–6]. Additionally, some evidence suggests the incidence of colorectal cancer may be reduced through the removal of precursor polyps and that the requirement for more invasive surgical procedures may be reduced due to earlier diagnosis [6].

A number of studies have attempted to characterise the inherent differences between screen-detected (SD) and non-screen-detected (NSD) cancer in terms of patient and tumour factors. Patients who have cancers detected at screening are more likely to be male, younger, less socioeconomically deprived have a lower systemic inflammatory response (SIR) and in those undergoing resection, to have lower T staging, less venous invasion, less peritoneal involvement and less margin involvement [7–9]. Co-morbidity is an important host factor that to date has not been studied in detail within the context of colorectal cancer screening outcomes. It has previously been shown that patients with screen-detected disease have a lower burden of co-morbidity due to their demographic profile, and that this may influence post-operative outcome [10]. However, the effect on long-term outcome and the potential for confounding by the SIR remains unclear. The aim of the present study was to assess the relationship between co-morbidity, screen-detection and overall survival in patients with colorectal cancer.

## Material and methods

A retrospective observational cohort study was conducted. The cohort was obtained from all patients invited to participate in the first complete round of the Scottish Bowel Screening Programme in NHS Greater Glasgow and Clyde (NHS GG&C) between April 2009 and the end of March 2011. As per the Scottish bowel cancer screening programme protocol, this involves those aged 50–74 years. To identify patients with non-screen-detected colorectal cancers diagnosed during the same time period and within the same health board, the West of Scotland Colorectal Cancer Managed Clinical Network (MCN) dataset and the Scottish Cancer Registry (SMR06) datasets were cross-referenced. In Scotland, colonoscopy is only routinely performed in asymptomatic individuals within the screening programme and so all non-screen-detected patients were scoped via symptomatic referral pathways. Further details on the identification of this cohort have previously been described [8].

Baseline demographics, co-morbidity, body mass index (BMI), American Society of Anaesthesiology grade (ASA) and survival were obtained on a case-by-case basis from NHS electronic patient records and theatre records. Patients were excluded from the final analysis if they did not undergo a surgical resection with curative intent or if their records were absent from the NHS electronic portal system.

Co-morbidity was objectively quantified using ASA and two validated co-morbidity scoring systems: the Lee Index and the Charlson Index. The American Society of Anaesthesiology grade is the gold standard system for assessing a patient’s preoperative physical status and medical co-morbidities and ranges from I for a normal healthy patient to V for a moribund patient not expected to survive with or without surgery. For the purposes of the analysis, an ASA grade of I-II was classified as low and an ASA of III-V as high. The Lee Index is a co-morbidity score which was developed to predict the risk of cardiac complications among patients undergoing non-cardiac surgery. It is based on six variables: a history of coronary artery disease, congestive heart failure, cerebrovascular disease, diabetes mellitus requiring insulin therapy, chronic kidney disease (defined as a preoperative serum creatinine >2 mg/dl) and whether the patient is due to undergo high-risk surgery (defined as intraperitoneal, intrathoracic or suprainguinal vascular surgery) [11]. As patients were only included in this study if they had undergone a colorectal resection, all patients scored at least 1 and a high Lee Index was defined as a score ≥2. The Charlson Index was developed to objectively quantify co-morbidity and associated mortality risk for the specific purpose of use in longitudinal studies. It is based on a history of myocardial infarction, congestive cardiac failure, peripheral vascular disease, cerebrovascular disease, dementia, chronic lung disease, connective tissue disease, peptic ulcer, diabetes mellitus (with or without end-organ damage), chronic kidney disease, hemiplegia, leukaemia, lymphoma, solid tumours (either localised or metastatic), liver disease (mild or moderate to severe) and AIDS [12]. A high Charlson Index was defined as a score ≥3.

Systemic inflammatory response (SIR) was quantified using the previously validated neutrophil/lymphocyte ratio (NLR). A greater SIR is associated with a high NLR (≥3).

## Statistics

Covariables were compared using crosstabulation and the *χ*^2^ test for a linear trend. A value of *P* < 0.05 was considered statistically significant. Overall, survival (OS) and cancer-specific survival (CSS) were analysed using Cox Regression. All covariables found to be statistically significant (*P* < 0.05) predictors of survival on univariate analysis were carried forward to a multivariate survival analysis. A stepwise backward method was used to produce a final model of variables with a significant independent impact on survival, where variables were removed from the model when the corresponding *P* value was >0.05. Statistical analysis was performed using SPSS software (SPSS Inc., Chicago, Illinois, USA).

## Results

### Participants

**Fig. 1:**
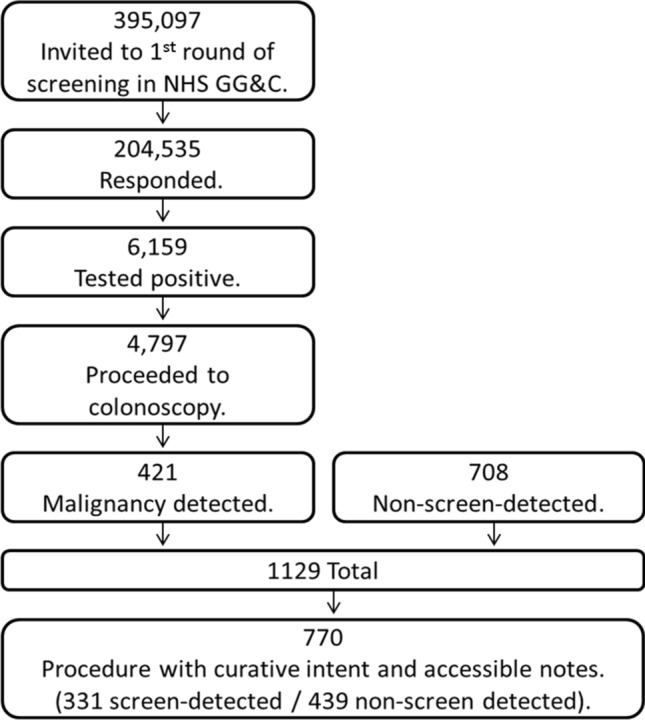
Flow chart of patient identification

Of all 395,097 patients invited to participate in the first complete round of screening in NHS GG&C, 204,535 (52%) responded of which 6,159 (3%) tested positive. Of those testing positive, 4797 (78%) proceeded to colonoscopy and 421 (9%) of those patients were found to have a colorectal malignancy. There were 708 patients with non-screen-detected colorectal cancers diagnosed in NHS GG&C during the same time period of which 468 (65%) of these were non-responders to the screening programme, 182 (25%) were interval cancers (within two years of a negative screening test), 43 (6%) were individuals who chose not to attend colonoscopy and 15 (2%) had no malignancy detected at index colonoscopy. Of the 1129 total (421 screen-detected and 708 non-screen-detected), 770 patients underwent a surgical resection with curative intent and had complete NHS electronic portal records and were included in the final analysis. A total of 331 (43%) of these patients had screen-detected (SD) and 439 (57%) had non-screen-detected (NSD) disease (Fig. 1).

## Demographics

Of all 770 patients included in the study, the median age was 67 years, 456 (59%) were males and 247 (37%) had rectal cancer. UICC-TNM distribution was stage I 234 (30%), II 262 (34%), III 236 (31%), IV 38 (5%). The SIR was elevated in 326 (43%) of all patients as measured by a high NLR (≥3).

A comparison of demographics between SD and NSD patients can be seen in Table 1. As has been reported previously in this cohort, patients with SD disease were significantly more likely to be male (64.4% vs 55.4%; *p* = 0.012), have an earlier UICC-TNM stage (*p* = 0.001), have colonic tumours (73.7% vs 63.4%; *p* = 0.002) and had a lower rate of emergency presentations (0.6% vs 17.1%; *p* < 0.001). Two (0.6%) screen-detected patients required emergency operations. The first was admitted for elective laparoscopic right hemicolectomy following positive screening, but on admission had clinical and radiological evidence of obstruction and perforation necessitating laparotomy and the second attended for colonoscopy following a positive screening test and was clinically and radiologically obstructed and was taken for an emergency subtotal colectomy. SD patients were less likely to have evidence of a SIR as measured by an elevated (≥3) NLR (33.7% vs 49.7%; *p* < 0.001).

**Table 1:** Baseline demographics and comparison of patients with screen-detected and non-screen-detected colorectal cancer

	All patients *n*(%)	Screen-detected *n*(%)	Non-screen-detected *n*(%)	*p* value
*Age*				0.259
≤62	254 (33.0%)	101 (30.5%)	153 (34.9%)	
63–70	256 (33.2%)	120 (36.3%)	136 (31.0%)	
≥71	260 (33.8%)	110 (33.2%)	150 (34.2%)	
*Sex*				**0.012**
Male	456 (59.2%)	213 (64.4%)	243 (55.4%)	
Female	314 (40.8%)	118 (35.6%)	196 (44.6%)	
*Scottish Index of Multiple Deprivation*				0.255
1 (most deprived)	254 (33.1%)	100 (30.3%)	154 (35.2%)	
2	141 (18.4%)	54 (16.4%)	87 (19.9%)	
3	129 (16.8%)	61 (18.5%)	68 (15.5%)	
4	107 (13.9%)	51 (15.5%)	56 (12.8%)	
5(least deprived)	137 (17.8%)	64 (19.4%)	73 (16.7%)	
*Presentation*				** <0.001**
Elective	693 (90.0%)	329 (99.4%)	364 (82.9%)	
Emergency	77 (10.0%)	2 (0.6%)	75 (17.1%)	
*Tumour Site*				**0.002**
Colon	521 (67.8%)	244 (73.7%)	277 (63.4%)	
Rectum	247 (36.6%)	87 (26.3%)	160 (36.6%)	
*UICC-TNM Stage*				**0.001**
I	234 (30.4%)	129 (39.0%)	105 (23.9%)	
II	262 (34.0%)	91 (27.5%)	171 (39.0%)	
III	236 (30.6%)	101 (30.5%)	135 (30.8%)	
IV	38 (4.9%)	10 (3.0%)	28 (6.4%)	
*ASA*^*a*^				**0.007**
Low (1–2)	439 (66.8%)	195 (72.8%)	244 (62.7%)	
High (≥3)	218 (33.2%)	73 (27.2%)	145 (37.3%)	
*Lee Index*				0.054
Low	620 (80.5%)	277 (83.7%)	343 (78.1%)	
High	150 (19.5%)	54 (16.3%)	96 (21.9%)	
*Charlson Index*				0.181
Low	577 (74.9%)	256 (77.3%)	321 (73.1%)	
High	193 (25.1%)	75 (22.7%)	118 (26.9%)	
*NLR*				** <0.001**
Low(<3)	435 (57.2%)	216 (66.3%)	219 (50.3%)	
High (≥3)	326 (42.8%)	110 (33.7%)	216 (49.7%)	

Significant *p* values highlighted in bold

*ASA* American Society of Anaesthesiology grade; *NLR* neutrophil/ lymphocyte ratio

^a^Data missing for 113 (14.7%) patients

## Co-Morbidity

Examining co-morbidity indices, SD patients were less likely to have a high (≥3) ASA score as compared to NSD patients (27.2% vs 37.3%; *p* = 0.007). There was no difference in the proportion of patients with a high (≥2) Lee Index (16.3% SD vs 21.9% NSD; *p* = 0.054) or high (≥3) Charlson Index score (23% SD vs 27% NSD; *p* = 0.181) between the groups.

## Survival

With a median follow-up of 63 months (range 33–83 months), 188 (24%) patients died of which 106 (56%) patients died of colorectal cancer. Eight (1%) died within 30 days of their operation (4 SD, 4 NSD). 5 year overall survival (OS) and cancer-specific survival (CSS) were 77% (168 deaths, 361 patients reaching 5 year follow-up) and 85% (100 deaths, 361 patients reaching 5 year follow-up), respectively.

**Fig. 2:**
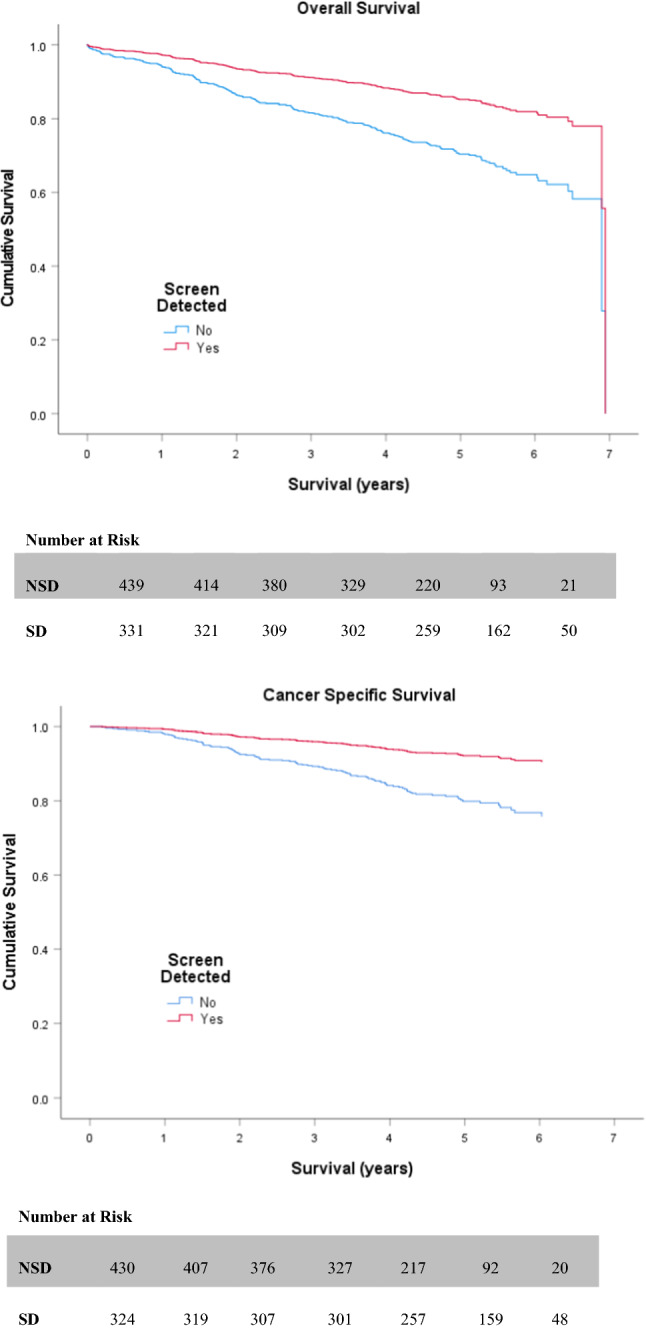
Relationship between screen detection and OS and CSS

**Fig. 3:**
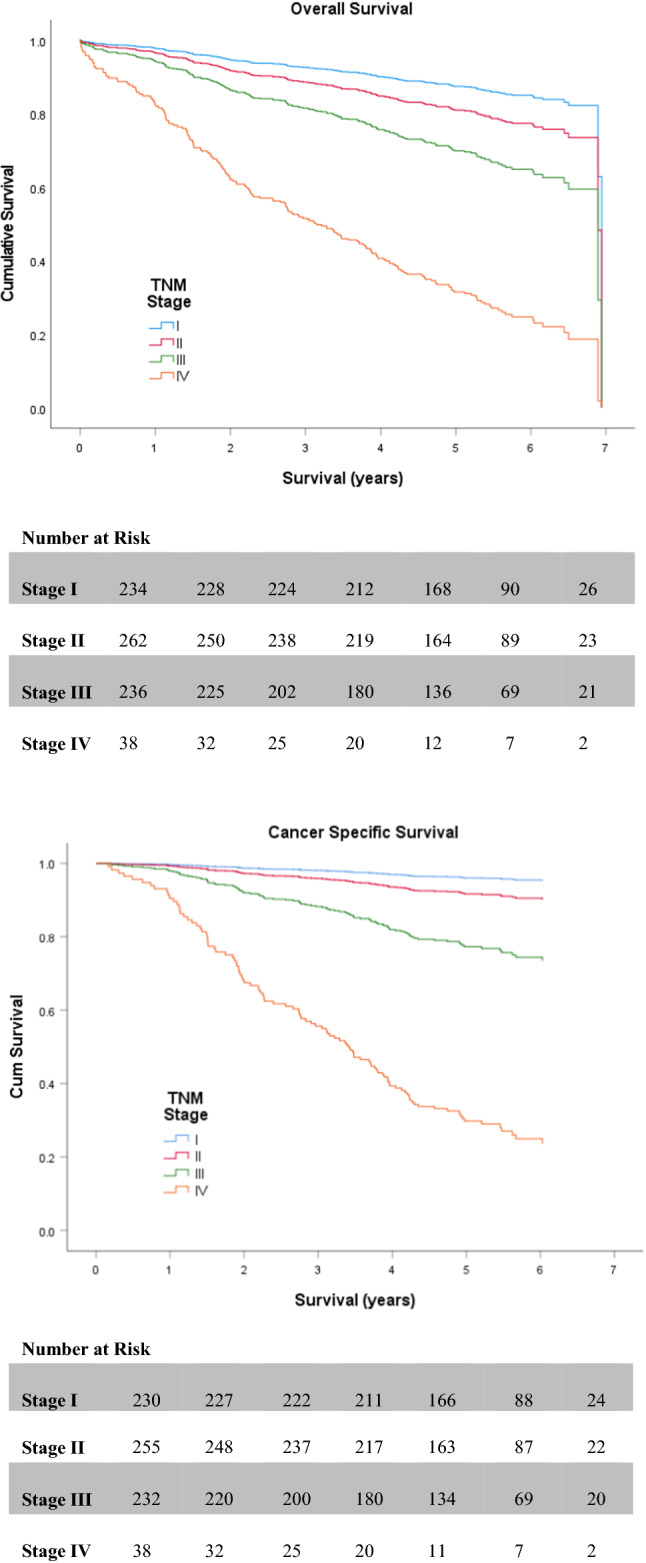
Relationship between UICC-TNM stage and OS and CSS

**Fig. 4:**
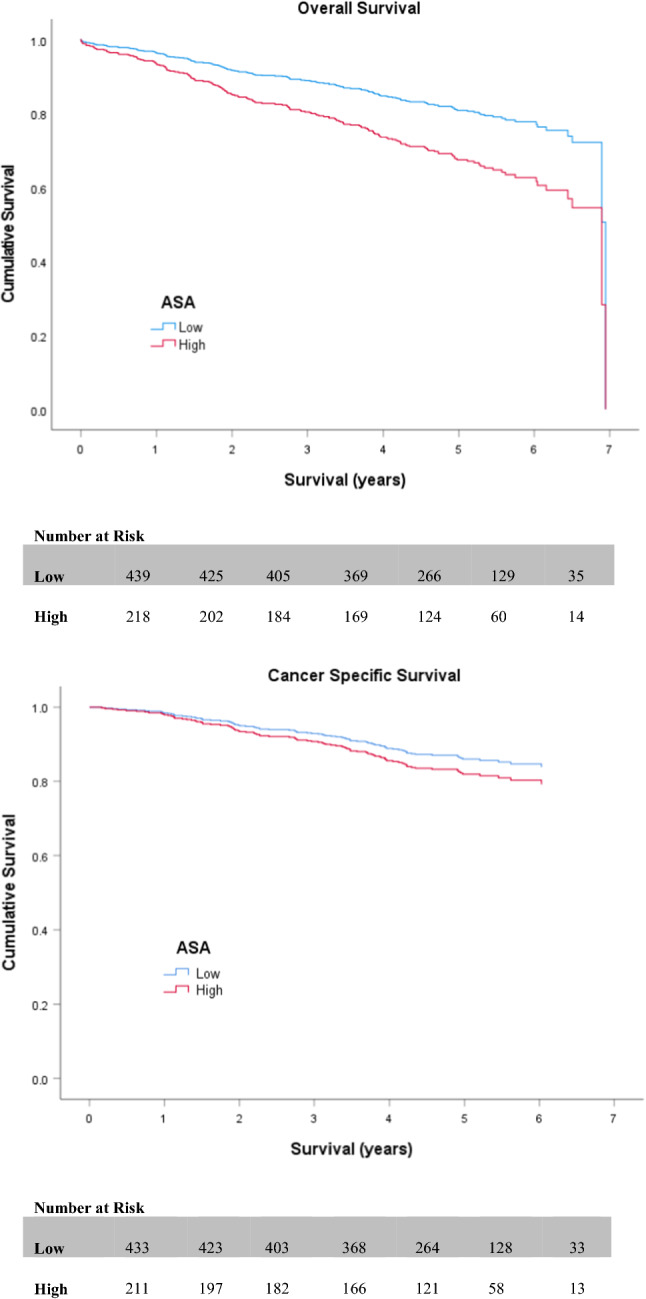
Relationship between ASA and OS and CSS

**Fig. 5:**
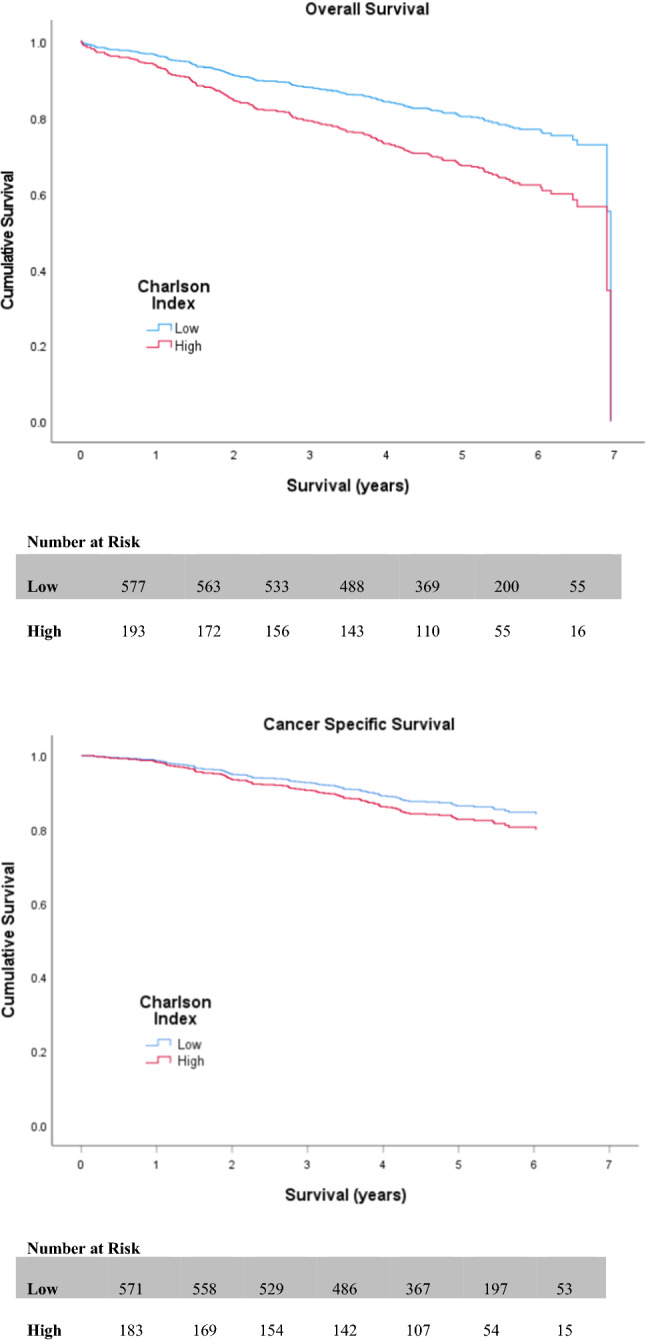
Relationship between Charlson Index and OS and CSS

**Fig. 6:**
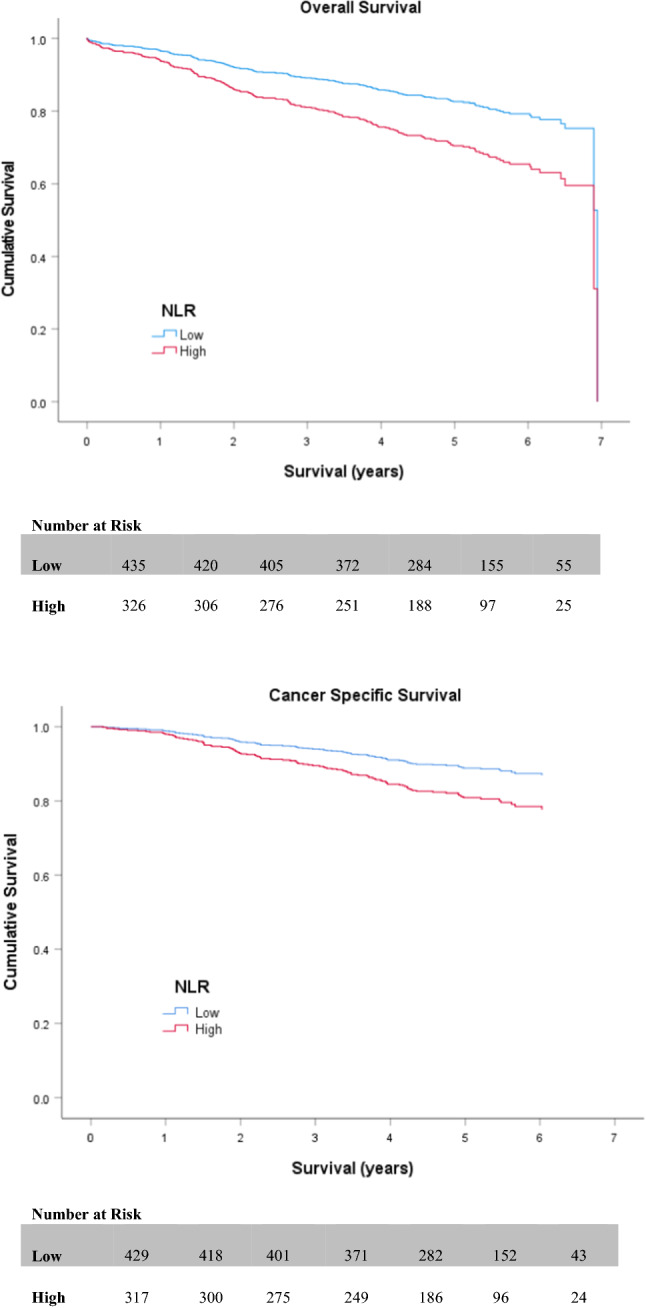
Relationship between Neutrophil/ Lymphocyte Ratio and OS and CSS

Table 2 and Table 3 display the outcomes of both univariate and multivariate survival analysis for OS and CSS, respectively. On univariate analysis, non-screen-detection (HR 2.182 (1.594–2.989; *p* < 0.001)) (Fig. 2), emergency presentation (HR 3.390 (2.401–4.788; *p* < 0.001)), advanced UICC-TNM stage (III or IV) (*p* < 0.001) (Fig. 3), high (≥3) ASA (HR 1.857 (1.362–2.532; *p* < 0.001)) (Fig. 4), high (≥3) Charlson Index (HR 1.800 (1.333–2.432; *p* < 0.001)) (Fig. 5) and high (≥3) NLR (HR 1.825 (1.363–2.442; *p* < 0.001)) (Fig. 6) were all associated with poorer overall survival. Excluding post-operative deaths, non-screen-detection (HR 2.763 (1.776–4.298; *p* < 0.001)), emergency presentation (HR 5.141 (3.388–7.801; *p* < 0.001)), advanced UICC-TNM stage (III or IV) (*p* < 0.001) and high (≥3) NLR (HR 1.793 (1.219–2.639; *p* = 0.003)) were also associated with poorer cancer-specific survival.

**Table 2:** Factors associated with overall survival in patients with colorectal cancer undergoing resection with a curative intent

	Univariate			Multivariable			
	H.R	95% C.I	*p* value	H.R		95% C.I	*p* value
*Age*							
<62	1.0						
63–70	1.075	0.743–1.555	0.702				
≥71	1.353	0.952–1.924	0.092				
*Sex*							
Male	1.0						
Female	0.968	0.721–1.299	0.828				
*Screen Detected*							
Yes	1.0			1.0			
No	2.182	1.594–2.989	** <0.001**	1.661	1.147–2.404		**0.007**
*SIMD*							
Non-deprived	1.0						
Deprived	1.242	0.931–1.658	0.141				
*Presentation*							
Elective	1.0			1.0			
Emergency	3.390	2.401–4.788	** <0.001**	2.190	1.451–3.304		** <0.001**
*Tumour Site*							
Colon	1.0						
Rectum	1.035	0.757–1.414	0.832				
*UICC-TNM Stage*							
I	1.0			1.0			
II	1.570	1.011–2.439	**0.045**	1.161	0.709–1.902		0.552
III	2.650	1.751–4.010	** <0.001**	2.374	1.501–3.755		** <0.001**
IV	8.567	5.147–14.261	** <0.001**	6.727	3.814–11.862		** <0.001**
*ASA*							
Low	1.0			1.0			
High	1.857	1.362–2.532	** <0.001**	1.260	0.893–1.778		0.189
*Lee Index*							
Low	1.0			1.0			
High	1.403	1.004–1.959	**0.047**	0.912	0.607–1.370		0.657
*Charlson Index*							
Low	1.0			1.0			
High	1.800	1.333–2.432	** <0.001**	1.732	1.240–2.421		**0.001**
*NLR*							
Low	1.0			1.0			
High	1.825	1.363–2.442	** <0.001**	1.271	0.933–1.794		0.122

Significant *p* values highlighted in bold

*ASA* American Society of Anaesthesiology grade. *NLR* neutrophil/ lymphocyte ratio

**Table 3:** Factors associated with cancer-specific survival in patients with colorectal cancer undergoing resection with a curative intent

	Univariate			Multivariable		
	H.R	95% C.I	*p* value	H.R	95% C.I	*p* value
*Age*						
<62	1.0					
63–70	0.741	0.461–1.189	0.214			
≥71	0.875	0.556–1.377	0.563			
*Sex*						
Male	1.0					
Female	1.036	0.702–1.528	0.859			
*Screen Detected*						
Yes	1.0			1.0		
No	2.763	1.776–4.298	** < 0.001**	1.924	1.193–3.102	**0.007**
*SIMD*						
Non-deprived	1.0					
Deprived	1.020	0.695–1.495	0.920			
*Presentation*						
Elective	1.0			1.0		
Emergency	5.141	3.388–7.801	** < 0.001**	2.557	1.608–4.067	** < 0.001**
*Tumour Site*						
Colon	1.0					
Rectum	1.208	0.787–1.853	0.388			
*UICC-TNM Stage*						
I	1.0			1.0		
II	2.153	0.980–4.730	0.056	1.585	0.714–3.521	0.258
III	6.405	3.149–13.027	** <0.001**	5.116	2.490–10.509	** < 0.001**
IV	30.064	14.054–64.313	** <0.001**	19.814	9.039–43.430	** < 0.001**
*ASA*						
Low	1.0					
High	1.321	0.868–2.009	0.194			
*Lee Index*						
Low	1.0					
High	1.245	0.784–1.977	0.354			
*Charlson Index*						
Low	1.0					
High	1.293	0.843–1.983	0.240			
*NLR*						
Low	1.0			1.0		
High	1.793	1.219–2.639	**0.003**	1.209	0.807–1.810	0.358

Significant *p* values highlighted in bold

*ASA* American Society of Anaesthesiology grade; *NLR* neutrophil/ lymphocyte ratio

On multivariable analysis non-screen-detection (HR 1.661 (1.147–2.404; *p* = 0.007)), emergency presentation (HR 2.190 (1.451–3.304; *p* < 0.001)), advanced UICC-TNM stage (III or IV) (*p* < 0.001) and a high (≥3) Charlson Index (HR 1.732 (1.240–2.421; *p* = 0.001)) retained significance as independent predictors of overall survival. Non-screen-detection (HR 1.924 (1.193–3.102; *p* = 0.007)), emergency presentation (HR 2.557 (1.608–4.067; *p* < 0.001)) and advanced UICC-TNM stage (III or IV) (*p* < 0.001) retained significance as independent predictors of cancer-specific survival.

## Discussion

The present study provides a comprehensive analysis of outcome in patients diagnosed with colorectal cancer following an invite to participate in the first round of the Scottish Bowel Screening Programme in our geographical area. It has identified that patients with screen-detected disease have tumours of an earlier UICC-TNM stage, have a lower systemic inflammatory response and have lower ASA scores than their non-screen-detected counterparts, one measure of co-morbidity. In addition, it has re-confirmed that having a screen-detected tumour is an independent prognostic factor for both improved overall and cancer-specific survival.

Many studies have attempted to characterise both factors that influence the uptake of bowel cancer screening and the inherent differences between screen-detected (SD) and non-screen-detected (NSD) cancer in terms of patient and tumour factors. For example, lower uptake of bowel screening has been shown to be associated with younger age, male sex and socioeconomic deprivation [7]. In agreement with previous work [7–9, 13], screen-detected patients in this study were more likely to be male, less likely to have rectal cancers and there was a non-significant trend towards lower socioeconomic deprivation. As would be expected, SD tumours were of a significantly lower UICC-TNM stage and there were a significantly lower number of emergency operations in this group. The NLR is a previously validated method of quantifying the systemic inflammatory response, with a higher NLR indicative of a greater SIR. NLR was significantly higher among NSD patients in this study.

The present study has added to existing work examining the degree of comorbidity in patients with SD compared to NSD colorectal cancer. Three measures of co-morbidity were used including the ASA, Lee Index and Charlson Index. Patients with SD disease were significantly less co-morbid as measured by the ASA. While a higher proportion of NSD patients had a high Lee Index co-morbidity score, this did not reach statistical significance (16.3% SD vs 21.9% NSD; *p* = 0.054). The reason behind this disparity in ASA scores is likely multifactorial and may reflect either the underlying difference in co-morbidity between those that choose to participate in the screening programme, or the morbidity associated with presenting with more advanced disease.

Indeed, the impact of co-morbidity on bowel cancer screening uptake has been previously studied [14]. This cross-sectional study which focussed on the Barcelona population-based colorectal cancer screening programme included 36,208 patients from 10 primary care centres with 17,404 (48%) of those participating in screening. Non-participants were significantly more likely to be male, socioeconomically deprived, smokers, have high-risk alcohol intake, be obese or be in the highest co-morbidity group. Having three or more dominant chronic diseases was associated with lower participation in the screening programme (incidence rate ratio IRR 0.76, 95% CI 0.65–0.89; *p* = 0.001) [14]. In addition, there is evidence that co-morbidity may be associated with non-participation in breast and cervical cancer screening programmes [15]. It is therefore conceivable that significant co-morbidity could act as a barrier to participating in the Scottish Bowel Screening Programme.

One previous study has examined the impact of screen-detection and co-morbidity on post-operative morbidity in patients undergoing resection for colorectal cancer. In this previous retrospective study from Spain of just under 200 patients, there was no significant difference between the SD and NSD groups in terms of ASA or Charlson Index, however, the percentage of patients with low ASA scores (I or II) was greater in the SD group [13].

The present study has a number of strengths related to its large numbers and level of detail regarding co-morbid disease. In addition, it is the first to report on the impact of the SIR on outcome in patients with screen-detected colorectal cancer. However, despite this, as a retrospective cohort study, ASA data were missing for 15% of patients. In addition, the effect of lead-time bias, where earlier detection artificially lengthens a patient’s survival following a cancer diagnosis, has not been taken into account. However, adjusting for this confounder within the context of a retrospective cohort study is complex and out with the scope of the present study.

## Conclusion

Patients with screen-detected disease have tumours of an earlier stage, have lower ASA scores and are less likely to have evidence of a SIR than their non-screen-detected counterparts. However, after adjusting for these co-variables, screen-detection retains significance as an independent predictor of improved overall and cancer-specific survival.

Therefore, yet undetermined inherent differences between SD and NSD patients remain and should be a focus of ongoing research.
